# Preparation, Characterization, and Application of *Ulva prolifera* Insoluble Dietary Fiber–Sodium Alginate–Cod Myofibrillar Protein Hydrogels for *Litopenaeus vannamei* Preservation

**DOI:** 10.3390/foods15081343

**Published:** 2026-04-13

**Authors:** Hao Wu, Han Zhang, Xu Zhao, Shu Liu, Jiayi Hu, Tiebin Wang, Song Gao, Guang Yang, Yaowei Fang

**Affiliations:** 1 Jiangsu Key Laboratory of Marine Bioresources and Environment, Co-Innovation Center of Jiangsu Marine Bio-Industry Technology, School of Marine Food and Biological Engineering, Jiangsu Ocean University, Lianyungang 222005, China; tefuirzy@163.com (H.W.); 19519620486@163.com (H.Z.); 19518833559@163.com (X.Z.); 2007000028@jou.edu.cn (S.L.); 18012420539@163.com (J.H.); gaos@jou.edu.cn (S.G.); 2020000050@jou.edu.cn (G.Y.); 2 Department of Animal Husbandry and Veterinary Medicine, Chifeng Vocational Institute of Applied Technology, Chifeng 024029, China; wangtb789@163.com

**Keywords:** cod protein, *Ulva prolifera* insoluble dietary fiber, *Litopenaeus vannamei* preservation

## Abstract

Background: Crosslinker-free, pH-induced hydrogels offer a green alternative for food preservation but often lack mechanical robustness. Objective: In this study, we developed a ternary hydrogel from cod myofibrillar protein (CP), sodium alginate (SA), and *Ulva prolifera*-derived insoluble dietary fiber (IDF) to enhance structural and preservation properties. Methods: Hydrogels with 0–3% IDF were characterized to assess their texture, water-holding capacity (WHC), and microstructure. Based on the balance between reinforcement and macroscopic processability, the 2% IDF formulation was selected for the shrimp preservation trial, which was conducted over 15 days at 4 °C. Results: Incorporation of 2% IDF significantly increased gel hardness (from 278.0 ± 6.8 g to 393.0 ± 1.8 g; *p* < 0.01, *n*^2^ = 0.87) and WHC (from 60% to 84.3 ± 2.1%; *p* < 0.01). In preservation tests, the CP-SA-IDF coating maintained TVB-N at 41.62 ± 3.7 mg/100 g, significantly lower than the control (78.65 ± 4.5 mg/100 g; *p* < 0.01) and inhibited microbial growth (TVC: 6.9 ± 0.3 log CFU/g vs. control 9.1 ± 0.4 log CFU/g; *p* < 0.05). A combined freshness index demonstrated superior overall preservation efficacy (0.82 vs. 0.49 in control; *p* < 0.05). Conclusions: IDF reinforces the CP-SA network via hydrogen bonding and physical entanglement, creating an effective edible coating for aquatic product preservation.

## 1. Introduction

With the growing emphasis on green and low-carbon strategies, natural hydrogels have garnered significant attention due to their biocompatibility, biodegradability, and tunable structural properties—attributes which are particularly valuable in cold-chain food preservation, biological sample storage, and low-temperature medical applications. These applications demand hydrogel materials with high structural stability, freeze–thaw resistance, and food-grade safety [1,2,3]. However, conventional hydrogels are typically produced through energy-intensive processes such as heat treatment, Ca^2+^ crosslinking, or chemical coupling. In food-related applications, some of these preparation routes, particularly those involving chemical reagents, may raise concerns regarding residue control and application safety, which limits their suitability for edible hydrogel systems [4]. Therefore, the development of composite hydrogels based on natural proteins and polysaccharides with enhanced environmental adaptability and safety has become a critical research focus [5].

Cod myofibrillar protein (CP), the primary protein fraction extracted from cod (*Gadus morhua*) muscle, is rich in myosin and actin. It is a high-molecular-weight protein with a molecular weight range of 40–220 kDa. CP has an isoelectric point (pI) of approximately 5.0, which is typical for myofibrillar proteins [6]. The protein contains several functional groups, including amine, carboxyl, and hydroxyl groups, contributing to its water retention, gelation, and emulsifying properties [7,8]. However, its low disulfide bond content leads to poor gel strength and structural integrity under freeze–thaw conditions. CP can form a network structure with insoluble dietary fiber (IDF) through physical entanglement and hydrogen bonding [7]. However, these binary systems often suffer from structural collapse and water loss under cold storage and exhibit limited long-term functional stability [8]. Sodium alginate (SA), a natural anionic polysaccharide, can form gels through pH-induced protonation of its carboxylate groups. Therefore, a ternary reinforcement mechanism needs to be developed to improve the structural robustness and environmental adaptability of such hydrogels.

Although many studies have focused on protein–polysaccharide hydrogels, they typically involve heat-denatured proteins such as whey or gelatin and rely on thermal or ionic gelation, which are less suitable for low-temperature systems [9,10]. A significant gap also exists in the development of crosslinker-free, pH-triggered ternary hydrogels for low-temperature preservation. Specifically, the integration of IDF as a natural, active reinforcing agent in such ternary systems, in which it can enhance low-temperature performance, remains largely unexplored [11]. In contrast, CP is a cold-water protein with a flexible conformation and few reactive sites, and its gelation behavior and freeze–thaw resilience remain poorly investigated. Moreover, systematic investigations on the use of natural physical crosslinkers to enhance low-temperature performance are still limited [12], particularly when it comes to concurrently enhancing mechanical robustness and barrier properties [13,14]. IDF, with its rigid framework and active surface groups (–OH, –COOH, and –SO_3_–), shows potential for hydrogen bonding, electrostatic attraction, and ionic complexation, contributing to network densification and water retention [15,16,17]. Marine-sourced IDF, particularly from *Ulva prolifera*, is abundant but has been studied for its potential adsorption, emulsification, or nutritional applications [18]. This combination of marine biomass valorization, physical reinforcement, and low-temperature-compatible gelation constitutes the main novelty of the present work. Although crosslinker-free ternary hydrogel systems have been reported previously, the present study differs in its use of *Ulva prolifera*-derived insoluble dietary fiber as a marine biomass-based physical crosslinking-assisted component in a relatively less explored cod myofibrillar protein/sodium alginate system. In this sense, the novelty of the present work lies not in the ternary format alone, but in the combination of composition selection, calcium-free gelation rationale, and preservation-oriented application [19,20].

In this study, we proposed a green gelation strategy based on “pH induction–IDF co-assembly” to fabricate a ternary hydrogel composed of CP, SA, and *U. prolifera*-derived IDF; to systematically evaluate the effects of IDF concentration on the appearance, water-holding capacity, texture, rheological behavior, thermal stability, and microstructure of the hydrogels; and to further assess the practical potential of the selected formulation for refrigerated shrimp preservation as a food-grade coating system. It can also promote the value-added utilization of cod byproducts and green tide algal resources. We hypothesized that IDF would exert a concentration-dependent effect on the CP-SA hydrogel system, and that an appropriate IDF level would improve gel performance and preservation efficacy.

## 2. Materials and Methods

### 2.1. Materials

*Ulva prolifera* (collected from the coastal region of the Yellow Sea, China; exact coordinates: [120°52′, 35°37′]) was used as the source of insoluble dietary fiber (IDF). Sodium alginate (SA, CAS No. 9005-38-3, viscosity: 180–220 mPa·s (1% in water, 25 °C), M/G ratio: 2:1) and D-(+)-gluconic acid δ-lactone (GDL, CAS No. 90-80-2) were used. The food-grade cod myofibrillar protein (CP) used was a commercial product purchased from Xi’an Xuhua Pharmaceutical Co., Ltd. (Xi’an, China). According to the manufacturer’s specifications, it is derived from cold-water cod, with a protein content of ≥90% (*w*/*w*), a molecular weight range of approximately 40–220 kDa, and an isoelectric point (pI) of 5.0. Thermostable α-amylase (500 U/g, CAS No. 9000-90-2), amyloglucosidase (30 U/g, CAS No. 9032–08-0), and protease (30 U/g, CAS No. 9014–01-1) were used.

### 2.2. Preparation of U. prolifera IDF

*Ulva prolifera* was washed four times with ddH_2_O (30:1, *v*/*w*), dried at 50 °C for 6 h, ground, and passed through a 40-mesh sieve. The powder was defatted and desugared; this involved rising it three times with 85% ethanol (10:1, *v*/*v*) for 2 min each and then drying it overnight at 40 °C. The resulting material was subjected to sequential enzymatic treatments to remove starch and protein. Specifically, it was treated with thermostable α-amylase (1:100, *w*/*w*, enzyme mass: substrate mass) in 50 mM maleic acid buffer at a solid-to-liquid ratio of 1:40 (*w*/*v*) at 95 °C for 35 min. This was followed by hydrolysis with amyloglucosidase (1:100, *w*/*w*, enzyme mass: substrate mass) at a solid-to-liquid ratio of 1:40 (*w*/*v*, in the same buffer) at 37 °C for 16 h, and subsequently with protease (1:100, *w*/*w*, enzyme mass: substrate mass) at a solid-to-liquid ratio of 1:40 (*w*/*v*) at 60 °C for 30 min. The final residue was collected via vacuum filtration, washed twice with hot water (70 °C), dried at 105 °C for 12 h, and ground to obtain IDF.

### 2.3. Preparation of Composite Hydrogels

Sodium alginate (SA; 2.5% *w*/*v*) and CP (3.0% *w*/*v*) were dissolved in ddH_2_O. The concentrations of sodium alginate (2.5%) and cod myofibrillar protein (3%) used in this study were selected based on preliminary trial experiments. SA emulsion: A total of 25 g of SA was dissolved in 1000 mL of sterile water, sonicated for 10 min, and stored at 4 °C. CP emulsion: CP (12 g) was dissolved in 400 mL of sterile water (pH 5) with 1 M HCl and stored at 4 °C. The SA and CP emulsions were mixed at a 3:4 (*v*/*v*) ratio, and IDF from *U. prolifera* was added at 1%, 2%, or 3% (*w*/*v*). After stirring for 5 min, GDL powder was added at 2% and 3% (*w*/*v*) and stirred for 10 min. Then, gelation was induced at 25 °C for 2 h, followed by storage at 4 °C overnight for setting. The information details are shown in Table 1.

### 2.4. Characterization of the Hydrogels

#### 2.4.1. Color Measurement

The color of the hydrogel was measured using a colorimeter (Model SC-10, 3nh, Shenzhen, China) against a white background. L* (lightness), a* (red-green), and b* (yellow-blue) values were recorded, with *n* = 3 biological replicates and 9 technical replicates per sample. Data are expressed as the mean ± SD [21].

#### 2.4.2. Water-Holding Capacity (WHC)

The hydrogels (4 °C, overnight) were weighed (M_0_, 2 g) and centrifuged at 5000 rpm for 30 min at 4 °C. Excess water was removed with filter paper and the final weight was recorded (M_1_) [22]. *n* = 3 biological replicates were used for each condition, and data are expressed as mean ± SEM. The WHC was calculated using the following equation:

WHC (%) = (M_1_/M_0_) × 100

Here, M_0_ indicates the initial gel mass (g) and M_1_ indicates the gel mass after centrifugation. Each sample was tested in quadruplicate.

#### 2.4.3. Texture Profile Analysis (TPA)

The textural properties were determined using a texture analyzer (TA-XT Plus, SMS, Surrey, UK). The samples, which were shaped into cylinders with a diameter of Φ18 mm and a height of 15 mm, were tested with a cylindrical probe (P/25). The probe penetrated 40% of the sample height at a speed of 1.0 mm/s, with a trigger force set at 5 g. Hardness, springiness, adhesiveness, chewiness, and cohesiveness were calculated from the force–time curves. *n* = 3 biological replicates were used for each treatment, and the data are expressed as mean ± SD. All tests were performed in triplicate [23].

#### 2.4.4. Gel Rheological Properties

Rheological behavior was assessed using a DHR-1 rheometer (TA Instruments, New Castle, DE, USA) with a 40 mm parallel plate geometry. The samples were pre-sheared at 100 s^−1^ for 1 min to remove bubbles and then allowed to rest for 2 min. Frequency sweep tests were performed at 1% strain over a 0.1–100 rad/s range at 25 °C. The shear viscosity was measured across a shear rate range of 1–100 s^−1^ [24]. These conditions were selected as standard small-amplitude rheological settings to characterize the intrinsic viscoelastic behavior of the hydrogel system and to enable reliable comparison among formulations. They were not intended to fully reproduce actual refrigerated storage or industrial processing conditions, but rather to provide a controlled basis for evaluating formulation-dependent structural differences.

#### 2.4.5. Fourier Transform Infrared (FTIR) Spectroscopy

The dried samples (5 mg) were mixed with KBr powder (100 mg), ground, and pressed into translucent pellets. FTIR spectra were obtained using a ReactIR 15 spectrometer (Mettler-Toledo, Greifensee, Switzerland) in the 4000–500 cm^−1^ range at a resolution of 4 cm^−1^ [25].

#### 2.4.6. X-Ray Diffraction (XRD)

X-ray diffraction (XRD) patterns were recorded to examine the effects of pH and the incorporation of IDF on the crystalline structure of the hydrogel. A Rigaku D/MAX 2500 diffractometer (Tokyo, Japan) with Cu Kα radiation was used, with a scan range of 2θ = 10–80° and a scan rate of 2°/min.

#### 2.4.7. Thermal Stability Analysis (TGA)

Thermal degradation behavior was assessed using a TGA 209F1 thermal analyzer (Netzsch, Selb, Germany). The dried hydrogel (10 mg) was heated from 30 °C to 600 °C at a rate of 20 °C/min under a stream of nitrogen (flow rate: 40 mL/min) [26].

#### 2.4.8. Scanning Electron Microscopy (SEM)

The microstructure of the freeze-dried hydrogels was analyzed using a scanning electron microscope (SU8010, Hitachi, Japan). The samples were gold-coated and observed at 300× magnification to examine their internal morphology [27].

### 2.5. Application of the Films in Shrimp Preservation

Fresh *Litopenaeus vannamei* shrimp were obtained directly from a local pond (location to be specified) and transported to the laboratory within 30 min in an ice-water slurry. After immobilization, the shrimp were rinsed with sterile water and selected for uniformity, with a body length of 10.5 ± 0.5 cm and a body weight of 4.2 ± 1.5 g. Only shrimp with intact appearance and without visible surface damage were included in the experiments. The surface moisture was removed, and the shrimp were randomly divided into three groups (blank, CP-SA, CP-SA-IDF) For the treatment groups, the shrimp were coated with CP-SA-IDF hydrogels as follows: the pre-gel emulsion was acidified with 1 M HCl to approach the isoelectric point, followed by the slow addition of GDL powder (2%, *w*/*w*). The mixture was stirred at room temperature for 30 min, during which the shrimp were immersed and evenly coated. After air-drying in a biosafety cabinet to form a uniform surface layer, the shrimp were sealed in sterile polyethylene bags and stored at 4 °C. The water activity (aw) of the shrimp was measured to assess the moisture level, and shrimp size and weight distribution were recorded. The samples in the control group were immersed in sterile water for 2 min. Samples were collected on days 0, 3, 6, 9, 12, and 15 for physicochemical analysis [28]. The storage conditions were carefully controlled to maintain specific temperature and relative humidity (4 ± 0.1 °C, 85 ± 1% RH) profiles throughout the experiment.

#### 2.5.1. pH Measurement

About 2.5 g of minced shrimp meat was homogenized with 25 mL of sterile water and left undisturbed for 30 min. The pH of the filtrate was measured at room temperature using a digital pH meter, with each sample measured in triplicate.

#### 2.5.2. Determination of Total Volatile Basic Nitrogen (TVB-N)

The sample (4.0 g) was homogenized with 20 mL of sterile water and extracted for 30 min. The filtrate (10 mL) was distilled into a flask containing 10 mL of 20 g/L boric acid solution and five drops of a mixed indicator (methyl red: bromocresol green = 1:5). After 5 min of steam distillation (plus 1 min with the condenser raised), the distillate was titrated with 0.01 M HCl or H_2_SO_4_ till the solution turned light purple [29].

#### 2.5.3. Total Viable Count (TVC)

Shrimp samples (10 g) were homogenized with 90 mL of sterile 0.85% saline and serially diluted 10-fold. Aliquots were spread on plate count agar (PCA) plates. Four to five dilutions were plated per sample, and counts were performed on plates with 30–300 colonies after incubation at 37 ± 1 °C for 48 ± 2 h. TVC was expressed as log CFU/g [21].

#### 2.5.4. Thiobarbituric Acid-Reactive Substances (TBAs)

Lipid oxidation was assessed using the TBA method. Briefly, 1 g of minced shrimp was combined with 9 mL of 7.5% trichloroacetic acid and incubated at 90 °C for 10 min. After cooling, the mixture was centrifuged at 5000 rpm for 10 min at 4 °C. The absorbance of the supernatant was measured at 532 nm. TBA values, expressed as mg MDA/kg, were calculated using a malondialdehyde (MDA) standard curve [30].

#### 2.5.5. Moisture Content and WHC

The moisture content was determined by drying 2–5 g of minced shrimp in pre-weighed flat-bottomed aluminum dishes (pre-dried at 105 °C for 1 h and cooled in a desiccator). The samples were dried at 105 °C for 2 h and then weighed. The drying cycle was repeated until the weight difference between the two measurements was less than 2 mg. The moisture content was calculated as follows:

The WHC was assessed using a method in which 3.0 g of shrimp mince (m_0_) was wrapped in filter paper and centrifuged at 8000 rpm for 10 min at 4 °C. The sample was weighed after centrifugation (m_1_), and the WHC was calculated as:

$$WHC\left(\%\right)=(1-\frac{m_{0}-m_{1}}{m_{0}})\times 100$$

#### 2.5.6. Combined Freshness Index (CFI)

The CFI was designed to integrate multiple preservation parameters (pH, TVB-N, TVC, MDA, MC, and WHC) into a single index, allowing for a comprehensive assessment of preservation performance. The parameters were first normalized to a range between 0 and 1 to account for their different units and scales. After normalization, we applied a weighted average of the parameters, where each parameter was assigned a weight based on its relative importance in preservation. The specific formula used to calculate the CFI is as follows:

$$\mathrm{CFI}\;=\;(\sum_{i=6}^{1}\omega_{i}\cdot N_{i})/(\sum_{i=6}^{1}\omega_{i})$$

where

N*_i_* is the normalized value of each parameter (pH, TVB-N, TVC, MDA, MC, and WHC). (The normalization of each parameter N*_i_* is done using the following formula: N*_i_* = (X_i_ − X_min_)/(X_max_ − X_min_). Where X_i_ is the raw value of the parameter for each sample. X_max_ and X_min_ are the minimum and maximum values observed across all samples for that parameter.)

ω*_i_* is the weight assigned to each parameter, based on its significance in evaluating preservation effectiveness. For example, we assign higher weights to microbial and nitrogen-related parameters like TVC and TVB-N, as they are more directly related to spoilage.

### 2.6. Statistical Analysis

The data were analyzed using SPSS 26.0 (IBM, Armonk, NY, USA) and visualized using OriginPro 2025 (OriginLab, Northampton, MA, USA). For the shrimp preservation experiment, two-way ANOVA was performed with treatment group and storage time (0, 3, 6, 9, 12, and 15 d) as fixed factors, and the treatment × storage time interaction was included in the model. Assumption checks (normality and homogeneity of variance) were conducted to ensure the validity of the ANOVA. For multiple comparisons, Tukey’s HSD post hoc test was used to control for type I error. Effect sizes (partial eta squared) and confidence intervals were reported to provide a more complete interpretation of the results. All differences were considered to be statistically significant at *p* < 0.05.

## 3. Results and Discussion

### 3.1. Composite Gel Appearance and Color

**Figure 1:**
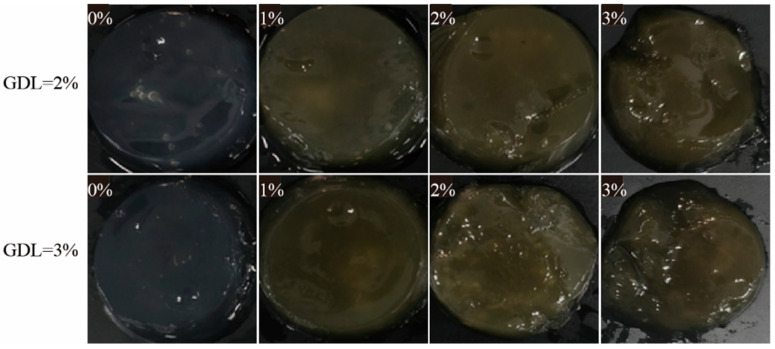
Appearance of composite gels with different IDF concentrations at 2% and 3% GDL.

To evaluate the reinforcing effect of IDF in the CP-SA system, hydrogels were prepared under various GDL concentrations (2% and 3%) and IDF additions (0–3%) (Figure 1). Gel morphology depended on both GDL and IDF concentration. At 2% GDL, the control gel (0% IDF) was opaque and shrunken, with a rough surface and evident syneresis. Adding 1% IDF improved shape regularity and noticeably smoothed the surface. The 2% IDF gel showed the best integrity in this series, presenting the most defined cylindrical form and the smoothest surface. However, further increasing the IDF to 3% impaired the structure, leading to collapse, surface cracks, and liquid release. A parallel trend was observed in the 3% GDL series (Figure 1), while the overall morphology was superior to that of the 2% GDL gels at the same IDF level. The 3% GDL control (0% IDF) was more coherent than its 2% GDL counterpart. The best formability and surface smoothness again occurred at 1–2% IDF. Notably, at 3% IDF, the gel formed with 3% GDL retained structural integrity far better than that formed with 2% GDL, although minor imperfections remained compared with the moderate-IDF groups. These observations indicate that IDF has a non-monotonic, concentration-dependent role in CP–SA gels. Moderate IDF (1–2%) likely reinforces the network by acting as a physical filler that improves network continuity and water immobilization, whereas excessive IDF (3%) introduces heterogeneity and defects that compromise the matrix. Increasing GDL to 3% promotes gel formation.

As shown in Table 2, at 2% GDL, L* decreased sharply after IDF addition (from 41.31 ± 0.89 at 0% to 25.02 ± 0.72 at 1%) and then rose slightly to 28.71 ± 0.46 at 3%. This pattern suggests reduced lightness with IDF incorporation, consistent with decreased surface light scattering due to improved matrix uniformity. Under 3% GDL, the L* range was narrower (21.19 ± 0.41–26.41 ± 0.34), indicating a more compact and color-stable gel structure at higher acidity. The a* values remained close to zero overall; gels at 2% GDL were nearly neutral (−0.16 ± 0.10 to 0.63 ± 0.10), whereas those at 3% GDL shifted slightly toward positive a*, which may relate to acid-induced protein aggregation affecting light reflection. In contrast, b* increased with IDF content, reaching 10.07 ± 0.78 at 2% GDL, indicating a stronger yellow hue with increasing IDF, particularly under acidic conditions [31,32].

Overall, CP–SA–IDF hydrogels showed a relatively dark yet visually uniform appearance with mild red/yellow tones. The color changes imply that IDF modifies optical properties and may be associated with network densification that alters light transmission. Given the importance of color uniformity for perceived quality, especially in seafood-like products [33], and the close link between structural uniformity, texture, and consumer trust IDF incorporation may support both appearance consistency and overall acceptability by reinforcing the CP–SA gel network [34].

### 3.2. Water-Holding Capacity

**Figure 2:**
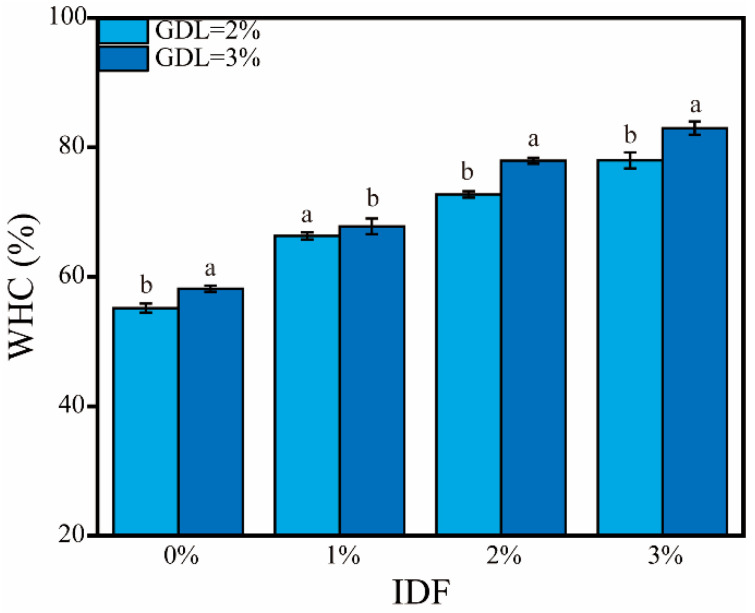
The WHC of composite gels with different IDF concentrations at 2% and 3% GDL. The data are expressed as the mean ± SD; *n* = 3. Different lowercase letters within the same GDL condition indicate significant differences among groups (*p* < 0.05).

The WHC is a critical parameter reflecting the ability of hydrogels to absorb and retain water, directly influencing texture retention, flavor release, and storage stability. The WHC of the CP-SA-IDF hydrogels increased significantly after IDF was added (*p* < 0.05) (Figure 2). At 2% GDL, the WHC increased from 58 ± 0.6% to 80 ± 1.2%, whereas at 3% GDL, it increased from 60 ± 0.4% to nearly 84.3 ± 2.1%, indicating that IDF effectively improved the water-binding ability of the gel. This improvement is likely related to (i) the porous structure of IDF, which provides capillary spaces that immobilize water, and (ii) abundant hydroxyl/carboxyl groups that promote hydrogen bonding and electrostatic association with CP and SA, thereby strengthening the gel network. At the same IDF level, WHC was consistently higher at 3% GDL than at 2% GDL, particularly at 2–3% IDF, suggesting that stronger acidification further enhances gelation and water immobilization, possibly via greater protein unfolding and ionic interactions [35]. Overall, CP–SA–IDF hydrogels showed excellent water retention under higher acidity with moderate IDF loading, supporting their potential in high-moisture biomimetic foods, nutrient delivery matrices, and edible moisture-retaining films. However, because no complementary analyses of swelling behavior or water mobility were performed in the present study, the present discussion should be interpreted as reflecting overall water-retention performance rather than detailed water-state dynamics.

### 3.3. Texture Profile Analysis

To assess the effect of IDF on the textural properties of CP-SA hydrogels, hardness, adhesiveness, springiness, cohesiveness, and chewiness were measured at IDF concentrations of 0–3% and GDL levels of 2% and 3% (Table 3). All parameters increased significantly after adding IDF (*p* < 0.05), with the most pronounced improvements observed at 3% GDL. In the 3% IDF group, the hardness reached 472.25 ± 2.99 g, whereas the springiness and chewiness were 1.02 ± 0.06 and 23.93 ± 0.19, respectively, which were substantially higher than those in the IDF-free group.

These results indicate that IDF not only serves as a physical filler but also enhances molecular entanglement through hydrogen bonding and electrostatic interactions between its hydroxyl/carboxyl groups and the CP-SA matrix, creating a more compact and ordered 3D structure. Compared with the IDF-free hydrogel, the improved structural recovery and textural performance under acid induction may be associated with enhanced CP-SA association and better integration of IDF within the network. However, direct evidence for CP conformational unfolding was not obtained in the present study, and this interpretation is therefore discussed only as a possible mechanism. The adhesiveness and cohesiveness also increased as the IDF content increased. At 2% IDF, adhesiveness reached 321.75 ± 7.41 N, and cohesiveness peaked at 0.89 ± 0.05 in the 3% IDF group, indicating enhanced interfacial interactions and energy dissipation. However, at 3% IDF, the decline in adhesiveness (Table 3) coupled with the observed macroscopic collapse (Figure 1) suggests that excessive fiber content increases interfacial friction and introduces structural defects, ultimately leading to network failure under stress. Taken together, these results suggest that the effect of IDF concentration was not strictly linear. Rather than showing a uniform monotonic improvement across all parameters, the system exhibited an optimal concentration range in which moderate IDF loading provided the best balance between mechanical enhancement, water retention, and macroscopic integrity, whereas excessive IDF impaired processability and structural stability [36].

This system achieves simultaneous improvements in elasticity and structural integrity via a fully physical route. Overall, IDF significantly improved the compressive strength, resilience, and structural rebuildability of hydrogels based on CP, making these hydrogels particularly suitable for high-moisture biomimetic seafood products or soft-solid delivery systems requiring strong rebound and textural retention [37,38].

### 3.4. Gel Rheological Properties

**Figure 3:**
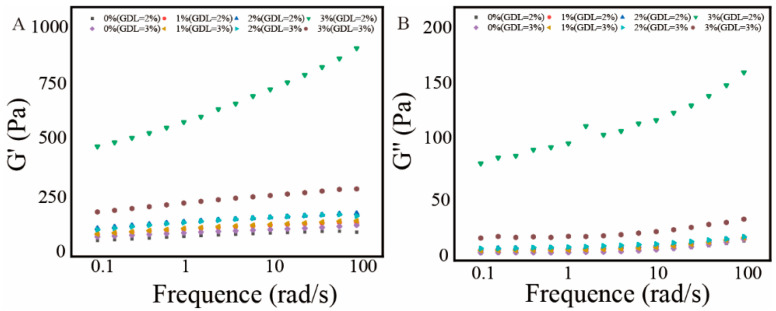
Variation in energy storage modulus (G′) and loss modulus (G″) composite gels with different IDF concentrations at 2% and 3% GDL. (**A**): storage modulus (G′); (**B**): loss modulus (G″).

Dynamic frequency sweep tests were performed to assess the viscoelastic properties of the CP-SA-IDF hydrogels by measuring the storage modulus (G′) and loss modulus (G″) across a frequency range of 0.1–100 rad/s. In all samples, G′ remained higher than G″ across the entire range, and both increased with frequency, indicating a predominantly elastic response and a weak gel behavior (Figure 3).

Adding IDF significantly enhanced viscoelasticity, particularly at mid-to-high frequencies (>10 rad/s). In the 3% IDF group, G′ increased considerably, reflecting improved structural rigidity and a greater energy storage capacity. A simultaneous increase in G″ suggested greater molecular friction and viscous dissipation, indicating enhanced damping behavior. Compared to the 0% IDF group, which presented a slower frequency response and looser structure, the IDF-filled hydrogels presented denser networks and stronger molecular entanglement, resulting in higher frequency sensitivity. CP is a marine-derived cold-water protein with high hydrophilicity and gel-forming ability. Under GDL induction, it formed electrostatic complexes with SA, whereas IDF further reinforced the network through pore filling, hydrogen bonding, and spatial anchoring. These interactions contributed to greater continuity and rigidity of the gel network, allowing structural recovery and resilience under shear [39,40,41].

Overall, the 3% IDF formulation presented the highest G′ and G″ values. From a practical perspective, the rheological results mainly reflect the relative structural stability and deformation resistance of the different formulations under controlled conditions, which is relevant to coating formation, handling, and structural maintenance. However, further rheological evaluation under refrigerated and application-specific processing conditions would be needed for direct industrial extrapolation.

### 3.5. Fourier Transform Infrared Spectroscopy

**Figure 4:**
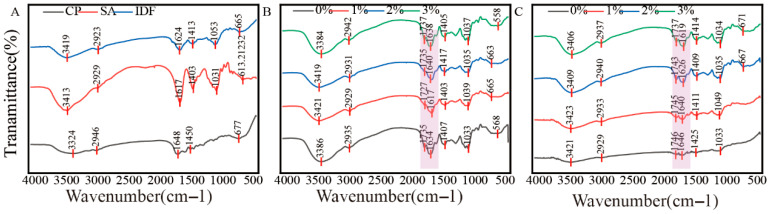
FTIR of composite gels with different IDF concentrations at 2% and 3% GDL. (**A**): CP, SA, and IDF. (**B**): 2% GDL; (**C**): 3% GDL.

Fourier transform infrared spectroscopy (FTIR) was performed to investigate the molecular interactions and functional group variations in CP-SA-IDF hydrogels (Figure 4). Broad –OH/–NH stretching bands were observed at 3280–3300 cm^−1^ in all individual components (CP, SA, and IDF), indicating the presence of abundant hydrogen bonding sites. SA showed characteristic COO^−^ asymmetric and symmetric stretching peaks at 1605 cm^−1^ and 1418 cm^−1^. The CP spectrum exhibited typical protein signatures, with the amide I band (primarily C=O stretch) located at around 1650 cm^−1^ and the amide II band (N–H bend/C–N stretch) near 1540 cm^−1^., whereas IDF exhibited C–O–C glycosidic vibrations at 1030–1070 cm^−1^, reflecting its typical cellulose backbone [42].

At 2% GDL, introducing IDF into the CP–SA gel produced several interaction-related changes. The –OH/–NH band (3280–3300 cm^−1^) showed a slight shift to lower wavenumbers and increased intensity, suggesting strengthened hydrogen-bonding associations. Meanwhile, the COO^−^ bands of SA exhibited minor shifts, indicating changes in the local electrostatic environment. Notably, variations in the shape and intensity of the IDF C–O–C region imply that cellulose domains are not simply dispersed but participate in interfacial association with the CP–SA matrix. Collectively, these spectral features suggest that IDF contributes to network formation beyond a passive filler role, consistent with the gel hardness (Table 3) and storage modulus G′ (Figure 3). At 3% GDL, under stronger acidic conditions, the spectral changes were more pronounced. At 3% GDL, the spectral changes became more evident. A weak band near 1730 cm^−1^ appeared, consistent with partial protonation of carboxylate groups (COO^−^ → COOH) under stronger acidification. In addition, more noticeable shifts in CP amide I/II bands suggest acid-induced conformational changes that may expose additional polar sites for interaction with SA and IDF. The C–O–C band around 1040 cm^−1^ also became sharper and/or shifted in the composites, especially at 3% IDF, indicating enhanced involvement and stabilization of cellulose-related structures within the gel network [43,44].

These FTIR observations generally align with the macroscopic and rheological results. The strengthened –OH/–NH band may imply increased polar association, which may contribute to both mechanical reinforcement and improved water binding, consistent with the higher WHC (Figure 2). Together with TPA (hardness/elasticity) and oscillatory rheology (G′), the data support a cooperative reinforcement process driven by acid-triggered structural adjustment of CP, multipoint polar association, and the spatial integration of cellulose domains in the composite network, although the specific interaction mode cannot be confirmed by FTIR alone [45].

### 3.6. X-Ray Diffraction

**Figure 5:**
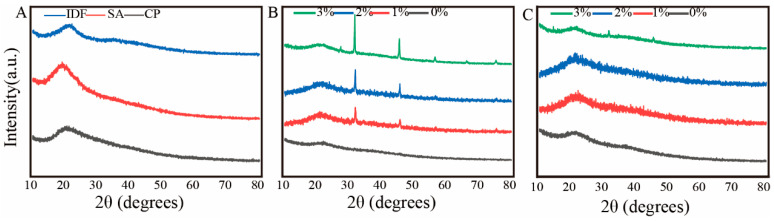
X-ray diffraction (XRD) patterns of composite gels with different concentrations of IDF at 2% and 3% GDL. (**A**): CP, SA, and IDF. (**B**): 2% GDL; (**C**): 3% GDL.

X-ray diffraction (XRD) was used to evaluate crystallinity and local ordering in CP–SA–IDF hydrogels under different acid induction conditions (Figure 5). IDF alone showed a broad peak at 2θ ≈ 22°, corresponding to the crystalline packing of cellulose microfibrils, whereas CP and SA exhibited broad, diffuse halos between 10° and 30°, indicating amorphous structures [19].

At 2% GDL, the IDF-related peak remained detectable in the composites, especially at 2–3% IDF, but with lower intensity than in pure IDF, suggesting partial embedding and/or reduced crystalline contribution of cellulose domains in the gel matrix. Weak additional signals (shoulders) were also observed, indicating the development of limited short-range ordering within the CP/SA matrix under mild acidification. At 3% GDL, the cellulose-related peak weakened further and was nearly absent in the 3% IDF gel, implying more extensive loss of microcrystalline order under stronger acid induction. The reduction in crystallinity suggests that cellulose domains become more integrated within the CP–SA network rather than remaining as independent crystalline regions. Such amorphization is commonly associated with greater network flexibility and improved water accommodation, which is consistent with the increased springiness/chewiness (Table 3) and higher WHC [46,47]. Notably, the samples with high GDL and high IDF showed the lowest crystallinity, yet they also exhibited the highest G′, hardness, and elasticity, indicating that reduced long-range order does not preclude mechanical strengthening. Instead, these results support a network dominated by dense physical associations and junction formation, where local interactions can enhance energy storage and resistance to deformation despite limited crystallographic order [48].

These findings support a cooperative reinforcement scenario in which acid induction promotes CP–SA association while IDF contributes through network integration and physical entanglement.

### 3.7. Gel Thermal Stability Analysis

**Figure 6:**
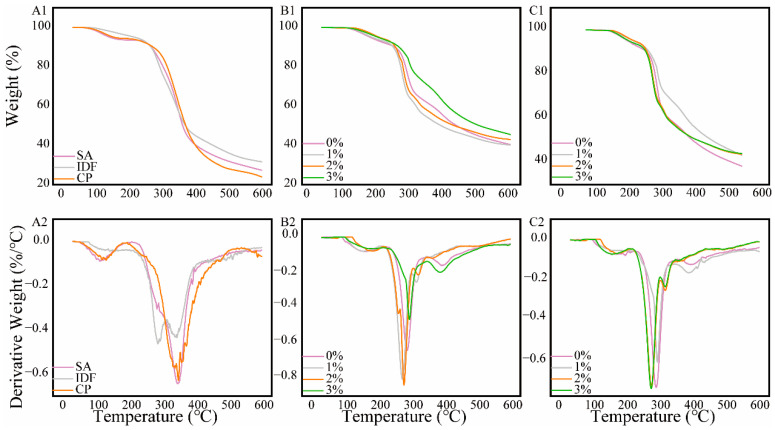
Thermogravimetric analysis (TGA) plots of composite gels with different concentrations of IDF at 2% and 3% GDL. (**A1**): TGA of IDF, CP, and SA. (**A2**): DTG of IDF, CP, and SA. (**B1**): TGA at 2% GDL. (**B2**): DTG at 2% GDL. (**C1**): TGA s at 3% GDL. (**C2**): DTG at 3%.

Thermogravimetric analysis (TGA) and derivative thermogravimetry (DTG) were conducted to evaluate the thermal degradation behavior of the CP-SA-IDF hydrogels over the temperature range of 25–600 °C. Among the individual components, IDF exhibited the highest T_max_ in DTG, indicating better thermal stability than SA and CP, which resulted in a major weight loss between 260 and 320 °C due to polysaccharide backbone and peptide chain breakdown (Figure 6).

At 2% GDL, all composite hydrogels displayed a three-stage profile: moisture loss (<150 °C), main decomposition of organics (220–340 °C), and gradual degradation of carbonaceous residues (>400 °C). Increasing IDF shifted T_onset_ and T_max_ to higher temperatures and increased the residual mass. The 3% IDF gel reached a T_max_ of 317.2 °C, suggesting improved thermal resistance. At 3% GDL, thermal stability increased further: the 3% IDF gel showed a T_max_ of 328.6 °C and a residue content approaching 30% at 600 °C. In addition, DTG peaks shifted to higher temperatures and decreased in intensity, indicating delayed decomposition and reduced thermal reactivity. The enhanced stability under higher GDL and IDF loading is consistent with a more consolidated network formed under stronger acid induction, with IDF contributing additional structural reinforcement. The increases in T_max_ and residue content are also consistent with the improved mechanical performance (hardness and G′), as a more thermally stable network typically reflects higher structural integrity. From an application perspective, a consolidated matrix may reduce component mobility during heating and storage, which is relevant for preservation-oriented systems [49,50].

### 3.8. Morphological Features

**Figure 7:**
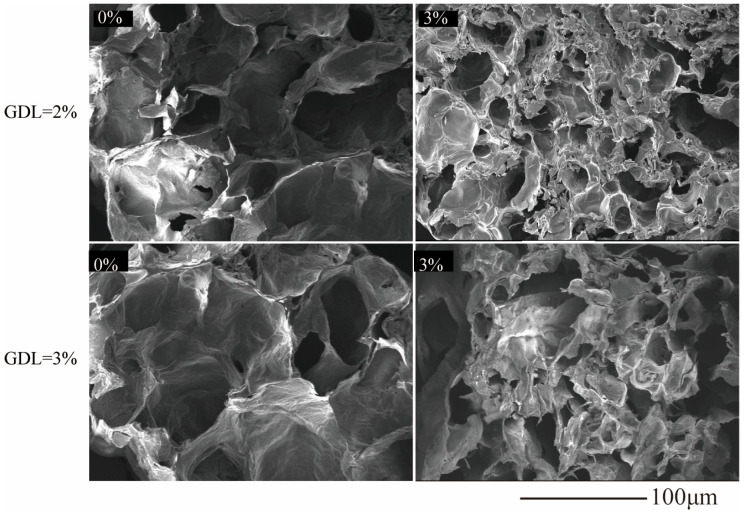
SEM of composite gels with different concentrations of IDF at 2% and 3% GDL.

Scanning electron microscopy (SEM) was performed to investigate the effect of IDF on the microstructure of the freeze-dried CP-SA hydrogels. In the absence of IDF, the hydrogels exhibited large, irregular pores with uneven distributions, thin pore walls, and localized collapse or rupture, indicating that the network structure was loose and unstable (Figure 7).

As the IDF content increased, the structure became more compact and honeycomb-like, with smaller pores, thicker walls, and improved continuity. This suggests that IDF promotes network consolidation, likely through physical reinforcement and interfacial association with the CP–SA matrix via its polar groups [51,52].

At 3% IDF, the gels exhibited the highest wall integrity and connectivity, which is consistent with the increased hardness/elasticity (TPA) and higher G′. Overall, SEM provides direct structural support for the reinforcing role of IDF and the suitability of CP–SA–IDF hydrogels for preservation films and biomimetic food matrices that require high structural integrity.

### 3.9. Comprehensive Evaluation and Determination of Optimal IDF Concentration

Based on the comprehensive characterization from macroscopic to microscopic levels (Section 3.1, Section 3.2, Section 3.3, Section 3.4, Section 3.5, Section 3.6, Section 3.7 and Section 3.8), a multi-objective trade-off analysis was conducted to definitively identify the optimal IDF concentration for the CP-SA hydrogel. The optimization criteria balanced mechanical performance (gel strength, G′, hardness), functional capacity (water-holding capacity), and critical processability (macroscopic structural integrity without syneresis or cracking, as shown in Figure 1).

The analysis reveals a clear dichotomy. On one hand, the 3% IDF formulation yields the peak values for storage modulus (G′) and hardness (Figure 3, Table 3), representing the ultimate mechanical reinforcement provided by the fiber. On the other hand, this mechanical zenith coincides with a catastrophic failure in macroscopic stability, as evidenced by pronounced syneresis, cracking, and structural collapse (Figure 1). This brittle and unstable behavior renders the 3% IDF hydrogel unprocessable for practical applications such as coating or molding.

In contrast, hydrogels incorporating 1–2% IDF exhibit a superior balance of properties. They possess significantly enhanced mechanical properties (substantially higher G′ and hardness than the control, Table 3, Figure 3), excellent water-holding capacity (Figure 2), and a uniform, compact microstructure (Figure 7), all while maintaining perfect macroscopic structural integrity and visual quality (Figure 7).

Therefore, it is conclusively determined that the optimal IDF concentration lies within the 1–2% range. Within this window, the marginal gain in mechanical properties observed at 3% IDF is decisively outweighed by the paramount requirement for macroscopic stability and processability. This optimized formulation was subsequently selected for the preservation application study.

### 3.10. Application of the Films in Shrimp Preservation

**Figure 8:**
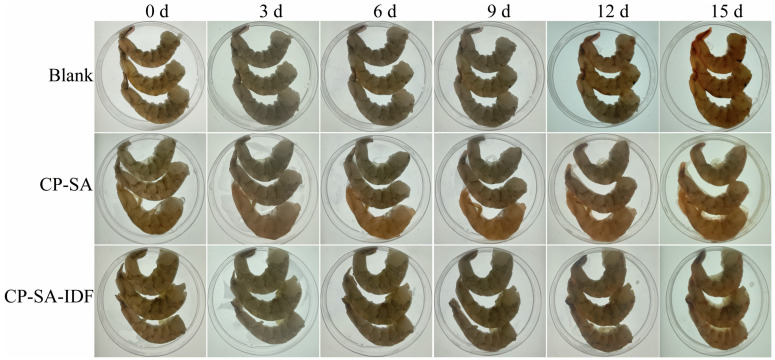
Appearance images of the shrimp coated with different concentrations of the gels during 15 days of storage.

During 15 days of refrigerated storage, the shrimp samples presented distinct changes in appearance, with prominent differences among the treatment groups. As shown in Figure 8, the blank group showed surface dehydration and browning by day 6; reddish-brown spoilage spots developed by day 12; and by day 15 the samples were dark brown, soft, and structurally degraded, indicating severe spoilage. The CP-SA group showed only slight improvement during days 6–9, but discoloration and loss of elasticity became evident after day 9; by day 15, pronounced browning and surface collapse were observed. In contrast, the samples in the CP–IDF group largely maintained their natural color and surface integrity throughout storage; even on day 15, only minor discoloration and edge darkening were observed, with no visible spoilage spots, resulting in markedly better sensory quality [53].

**Figure 9:**
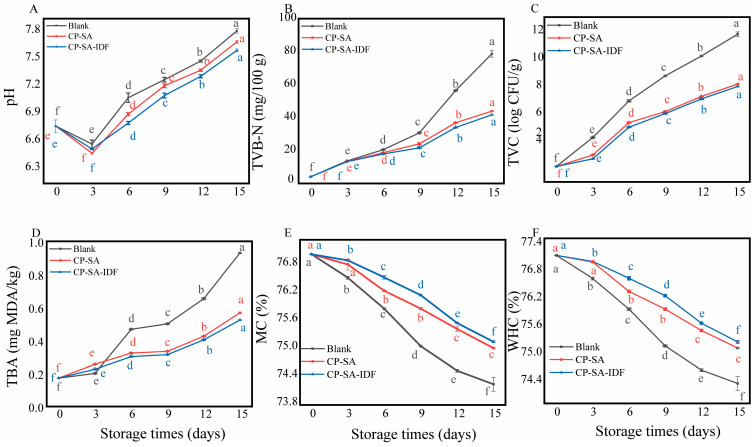
Changes in pH (**A**), TVB-N (**B**), TVC (**C**), TBA (**D**), MC (**E**), and WHC (**F**) of shrimp during storage. The data are expressed as the mean ± SD; *n* = 3. Any group containing one identical lowercase letter indicates a non-significant difference (*p* < 0.05).

To provide an integrated assessment of preservation performance and avoid over-reliance on any single index, a combined freshness index (CFI) was calculated at day 15 by normalizing and averaging six parameters (pH, TVB-N, TVC, MDA, total moisture content, and WHC). The CP–IDF group achieved the highest CFI (0.82 ± 0.01), significantly higher than CP-SA (0.71 ± 0.02) and the blank (0.49 ± 0.04) (*p* < 0.05), indicating the best overall freshness retention. This ranking was consistent with day-15 endpoints across key quality dimensions (Figure 9): pH was lowest in CP–IDF (7.44 ± 0.03) versus CP-SA (7.62 ± 0.02) and blank (7.84 ± 0.04) indicating a more effective suppression of spoilage-associated alkaline metabolites; TVC was reduced to 6.9 ± 0.4 log CFU/g in CP–IDF compared with 7.6 ± 0.4 (CP-SA) and ~9 ± 0.3 (blank); MDA was lowest in CP–IDF (1.43 ± 0.03 nmol/mg) versus CP-SA (1.68 ± 0.04) and blank (2.10 ± 0.04); and moisture/WHC were better maintained in CP–IDF (75.1 ± 0.4% and 74.3 ± 0.4%) than in the blank (73.4 ± 0.4% and 72.6 ± 0.3%). TVB-N increased in all groups, with the blank reaching 78.65 ± 4.5 mg/100 g (spoilage level). After correction, the CP-SA-IDF group showed a lower TVB-N value than the CP-SA group at the final sampling point, which was consistent with it having the highest CFI and better overall preservation performance [54].

Overall, the CP-SA–IDF coating provided the most balanced preservation, as supported by it achieving the highest CFI (0.82). It slowed pH rise, stabilized TVB-N changes, inhibited microbial growth, reduced lipid oxidation, and improved water retention. As a fully physical, food-grade system, this composite film offers a practical and green strategy for preserving high-moisture aquatic products [55].

## 4. Conclusions

An edible CP–SA–IDF hydrogel was formed by GDL-induced acidification in a fully physical, crosslinker-free system. IDF increased network compactness and improved G′/hardness and thermal stability, but excessive IDF compromised macroscopic integrity. Although 3% IDF produced the highest G′ and hardness, it caused syneresis, cracking, and collapse; 2% IDF provided the best balance of reinforcement and processability and was therefore selected for application. Structural results supported IDF–matrix reinforcement mainly via noncovalent association (hydrogen bonding and electrostatic interactions). In refrigerated shrimp storage, the CP–SA-IDF film slowed pH rise, TVB-N accumulation, and lipid oxidation, improved water retention and sensory quality, and extended shelf life by 3–5 days versus both uncoated and CP-SA controls. This system offers a practical route for seafood preservation and high-value utilization of green-tide biomass. However, this study was conducted at laboratory scale with limited replication, and the preservation performance was evaluated only under one refrigerated condition using a single aquatic product model. In addition, the reinforcement mechanism was inferred mainly from indirect evidence, and scale-up validation was not performed. It should be noted that the present IDF extraction procedure was established at laboratory scale. Although it was effective for preparing IDF for the current study, the multiple enzymatic, washing, and drying steps involved may increase processing complexity, time, and cost during scale-up. Future work should focus on expanded replication, validation in additional aquatic products and storage scenarios, direct mechanistic characterization, and pilot-scale evaluation of coating feasibility.

## Figures and Tables

**Table 1:** Formulation and processing parameters for CP-SA-IDF hydrogel preparation.

Parameter	Value/Range
IDF concentration (%)	1%, 2%, 3%
GDL concentration (%)	2%, 3%
Total solids content (%)	5%
Mixing/shear conditions	Stirring at 300 rpm for 10 min
pH trajectory	pH 7.0 → pH 5.0
Gelation time–temperature profile	25 °C for 2 h, followed by overnight storage at 4 °C

**Table 2:** Color of composite gels with different IDF concentrations at 2% and 3% GDL.

	Sample	L*	a*	b*
GDL = 2%	0%	41.31 ± 0.89 ^a^	0.63 ± 0.10 ^a^	6.83 ± 0.28 ^c^
	1%	25.02 ± 0.72 ^d^	0.25 ± 0.04 ^b^	6.33 ± 0.25 ^d^
	2%	26.75 ± 1.03 ^c^	−0.16 ± 0.10 ^c^	8.92 ± 0.56 ^b^
	3%	28.71 ± 0.46 ^b^	0.56 ± 0.26 ^a^	9.89 ± 0.67 ^a^
GDL = 3%	0%	26.41 ± 1.06 ^a^	1.14 ± 0.03 ^a^	10.63 ± 0.22 ^a^
	1%	21.19 ± 0.41 ^c^	0.31 ± 0.06 ^c^	5.62 ± 0.48 ^c^
	2%	25.16 ± 0.86 ^b^	0.14 ± 0.22 ^d^	9.48 ± 0.94 ^b^
	3%	26.34 ± 0.34 ^a^	0.60 ± 0.11 ^b^	10.07 ± 0.78 ^ab^

Note: The data are expressed as the mean ± SD; *n* = 3. Different lowercase letters within the same GDL condition indicate significant differences among groups (*p* < 0.05).

**Table 3:** Texture profile analysis of composite gels with different IDF concentrations at 2% and 3% GDL.

	Sample	Hardness (gf)	Adhesiveness (N)	Springiness	Cohesiveness	Chewiness
GDL = 2%	0%	136.50 ± 2.38 ^d^	81.75 ± 4.35 ^d^	0.01 ± 0.01 ^d^	0.18 ± 0.01 ^d^	2.33 ± 0.02 ^d^
	1%	216.00 ± 5.35 ^c^	113.75 ± 4.11 ^c^	0.09 ± 0.01 ^c^	0.27 ± 0.02 ^c^	11.54 ± 0.03 ^c^
	2%	290.50 ± 4.43 ^b^	150.50 ± 4.04 ^b^	0.23 ± 0.01 ^b^	0.48 ± 0.01 ^b^	12.35 ± 0.04 ^b^
	3%	358.50 ± 2.08 ^a^	247.00 ± 3.92 ^a^	0.78 ± 0.02 ^a^	0.66 ± 0.01 ^a^	15.32 ± 0.03 ^a^
GDL = 3%	0%	278.00 ± 6.78 ^d^	136.00 ± 2.45 ^d^	0.04 ± 0.01 ^d^	0.41 ± 0.02 ^d^	4.70 ± 0.05 ^d^
	1%	352.00 ± 2.94 ^c^	247.00 ± 11.69 ^c^	0.12 ± 0.01 ^c^	0.54 ± 0.02 ^c^	16.57 ± 0.31 ^c^
	2%	393.00 ± 1.83 ^b^	321.75 ± 7.41 ^a^	0.33 ± 0.02 ^b^	0.81 ± 0.03 ^b^	19.68 ± 0.22 ^b^
	3%	472.25 ± 2.99 ^a^	283.00 ± 3.74 ^b^	1.02 ± 0.06 ^a^	0.89 ± 0.05 ^a^	23.93 ± 0.19 ^a^

Note: The data are expressed as the mean ± SD; *n* = 3. Different lowercase letters within the same GDL condition indicate significant differences among groups (*p* < 0.05).

## Data Availability

The original contributions presented in this study are included in the article. Further inquiries can be directed to the corresponding author.
